# Sulodexide for the Prevention of Recurrent Venous Thromboembolism: The Sulodexide in Secondary Prevention of Recurrent Deep Vein Thrombosis (SURVET) Study: A Multicenter, Randomized, Double-Blind, Placebo-Controlled Trial

**DOI:** 10.1161/CIRCULATIONAHA.115.016930

**Published:** 2015-11-16

**Authors:** Giuseppe M. Andreozzi, Angelo A. Bignamini, Giovanni Davì, Gualtiero Palareti, Jiří Matuška, Martin Holý, Katarzyna Pawlaczyk-Gabriel, Andrej Džupina, German Y. Sokurenko, Yury P. Didenko, Laurentia D. Andrei, Gianfranco Lessiani, Adriana Visonà

**Affiliations:** From Angiology Unit, University Hospital, Padua, Italy (G.M.A.); School of Specialization in Hospital Pharmacy, Department of Pharmaceutical Sciences, University of Milan, Italy (A.A.B.); Department of Medicine and Aging, “G. D’Annunzio” University, Chieti, Italy (G.D., G.L.); Cardiovascular Diseases, University of Bologna, Italy (G.P.); Angiologická Ambulance, Hodonín, Czech Republic (J.M.); Nemocnice České Budějovice–Interní Oddělení, České Budějovice, Czech Republic (M.H.); Department of Hypertensiology, Angiology and Internal Diseases, Medical University, Poznań, Poland (K.P.-G.); ALIAN s.r.o., Bardejov, Slovakia (A.D.); Federal State Institute, Nikiforov Russian Center of Emergency and Radiation Medicine, St. Petersburg, Russia (G.Y.S.); St. Petersburg State Medical Institution, City Multidisciplinary Hospital No. 2, Russia (Y.P.D.); Spitalul Clinic Judetean de Urgenta, Braşov, Romania (L.D.A.); and Angiology Unit, Hospital of Castelfranco Veneto, Italy (A.V.).

**Keywords:** glycosaminoglycans, randomized controlled trial, recurrence, venous thromboembolism

## Abstract

Patients with a first episode of unprovoked venous thromboembolism have a high risk of recurrence after discontinuation of anticoagulant therapy. Extending anticoagulation reduces the risk of recurrence but is associated with increased bleeding. Sulodexide, a glycosaminoglycan, exerts antithrombotic and profibrinolytic actions with a low bleeding risk when administered orally, but its benefit for preventing recurrent venous thromboembolism is not well known.

**Methods and Results—:**

In this multicenter, double-blind study, 615 patients with first-ever unprovoked venous thromboembolism who had completed 3 to 12 months of oral anticoagulant treatment were randomly assigned to sulodexide 500 lipasemic units twice daily or placebo for 2 years, in addition to elastic stockings. The primary efficacy outcome was recurrence of venous thromboembolism. Major or clinically relevant bleeding was the primary safety outcome. Venous thromboembolism recurred in 15 of the 307 patients who received sulodexide and in 30 of the 308 patients who received placebo (hazard ratio, 0.49; 95% confidence interval [CI], 0.27–0.92; *P*=0.02). The analysis in which lost to follow-up was assigned to failure yielded a risk ratio among treated versus control subjects of 0.54 (95% confidence interval, 0.35–0.85; *P*=0.009). No major bleeding episodes occurred; 2 patients in each treatment group had a clinically relevant bleeding episode. Adverse events were similar in the 2 groups.

**Conclusion—:**

Sulodexide given after discontinuation of anticoagulant treatment reduced the risk of recurrence in patients with unprovoked venous thromboembolism, with no apparent increase of bleeding risk.

**Clinical Trial Registration—:**

URL: https://www.clinicaltrialsregister.eu/. Identifier: EudraCT number 2009-016923-77.

## Abstract 2

Supplemental Digital Content is available in the text.

The risk of recurrence of venous thromboembolism (VTE) persists for many years after anticoagulant treatment is withdrawn^1^ and is particularly high among patients with unprovoked VTE.^2^ About 20% of patients have a recurrence within 2 years after discontinuation of treatment with a vitamin K antagonist (VKA).^3–6^ Extending the treatment with VKA reduces the risk of recurrence but increases the risk of bleeding, as well as the inconvenience and costs of laboratory monitoring and dose adjustments.^7,8^ The effects of the newer non-VKA oral anticoagulants for therapy of acute VTE events^9–12^ and for extended treatment to avoid recurrences^13,14^ have recently been investigated by a number of clinical trials that, as a whole, showed an efficacy noninferior to VKA and rates of bleeding in general inferior to VKA, especially for extended treatment.

Editorial see p 1856

Clinical Perspective on p 1897

Sulodexide is a natural glycosaminoglycan with antithrombotic and profibrinolytic activities^15^ that can be administered orally or parenterally and affects the normal hemostasis to a lower extent than heparin with a very low risk of bleeding. Several clinical studies proved that prolonged sulodexide administration was associated with no or negligible risk of bleeding,^16–18^ as also highlighted in a recent review.^19^ Sulodexide exerts its actions through complexation with antithrombin and heparin cofactor II and the attending inhibition of some factors of the coagulation cascade.^20–22^ It also exerts favorable effects on endothelial dysfunction, release of cytokines and chemokines, and metalloprotease-9 secretion from white blood cells.^23,24^

The pharmacological and clinical profiles suggest that oral sulodexide may have a role in the prevention of recurrent VTE when classic anticoagulation is discontinued. Indeed, recent clinical studies proved a positive effect of oral sulodexide administration in reducing the risk of recurrence compared with either anticoagulation with acenocoumarol^25^ or standard of care after withdrawal of VKA treatment.^18^ The aim of this randomized, double-blind, controlled trial (Sulodexide in Secondary Prevention of Recurrent Deep Vein Thrombosis [SURVET]) was to verify the efficacy and safety of sulodexide in the prevention of recurrent VTE after the end of the VKA treatment in patients with a first-ever unprovoked VTE.

## Methods

### Patients

We recruited patients of ≥18 years of age with a documented first-ever unprovoked proximal deep vein thrombosis or pulmonary embolism treated with VKA for 3 to 12 months. VTE was considered unprovoked when it occurred in the absence of any known risk factor for this event. We excluded patients with persistent pulmonary hypertension after pulmonary embolism, those with solid neoplasm or blood disease, those with anti-phospholipid antibody syndrome or antithrombin congenital deficit, patients with New York Heart Association class III to IV cardiorespiratory failure, and patients with known hypersensitivity to glycosaminoglycans. Fertile women were enrolled if not lactating if their pregnancy test at screening was negative and they were willing to use contraception (except oral contraceptives) throughout the study period. Each subject was enrolled only after having issued the written informed consent to participate to the study.

### Study Design and Intervention

SURVET was a multicenter, multinational, randomized, double-blind, parallel-group, placebo-controlled clinical trial. Eligible patients were allocated to treatment for 2 years with oral sulodexide (2×250–lipasemic unit capsules twice daily) or matching placebo in a 1:1 ratio based on a computer-generated randomization list in blocks of 4 produced by an independent operating unit. This same unit also packaged drug and matching placebo in identical-looking treatment units, 1 for each randomized patient, identified exclusively by the randomization number. Patients, recruiting physicians, physicians or pharmacists delivering the treatments units, physicians or technicians assessing the outcome, and Steering Committee members were blinded to the intervention and to the block size until the end of the statistical analysis. Each sequentially numbered treatment unit was accompanied by an opaque, sealed envelope that allowed unblinding of the individual patient treatment in case of need. Randomization occurred within 1 to 12 weeks after VKAs had been withdrawn, with the patient assigned to the treatment unit with the lowest number available at the relevant study center.

### Outcome Measures

The central adjudication committee members who were unaware of the group assignments and who reviewed all the patients’ raw data assessed all suspected study outcome events. The primary efficacy outcome was symptomatic, objectively confirmed recurrence of VTE, defined as the composite of deep vein thrombosis objectively confirmed by compression ultrasonography^26^ and nonfatal or fatal pulmonary embolism objectively confirmed by computed tomography or lung scanning. Secondary efficacy outcomes included distal or superficial vein thrombosis and nonfatal or fatal myocardial infarction, stroke, or acute ischemia of the lower limbs.

The principal safety outcome was major or clinically relevant nonmajor bleeding. An overt bleeding event was defined as major if fatal, if it occurred in a critical location, or if it required a transfusion of ≥2 U whole blood or red cells. Clinically relevant nonmajor bleeding was defined as overt bleeding that did not meet the criteria for major bleeding but was associated with the need for medical intervention, contact with a physician, interruption of the study drug, or discomfort or impairment of activities of daily life.^27^

### Surveillance and Follow-Up

The investigators, according to the study protocol, recommended to each participant the use of a class II elastic stocking after the diagnosis of proximal deep vein thrombosis. Their use was to be continued for 2 years. The investigators renewed this recommendation at each periodic visit. Patients were re-examined at the relevant clinical center every 3 months for 24 months after randomization. Patients were instructed to report to the study center if they had symptoms suggestive of VTE, other circulatory events, or bleeding complications for objective evaluation. Each patient was contacted by telephone every month between examinations. In case of symptoms suggesting that an end point occurred, the patient was invited to the center of reference for an unplanned interview. Symptoms and signs suggestive of adverse events (AEs) were also recorded. At month 24, we contacted by telephone all patients who prematurely interrupted or left the study without formally withdrawing consent so that we could monitor whether symptoms or signs suggestive of a vascular event had occurred.

### Study Oversight

The members of the Steering Committee designed the study, registered in the EU Clinical Trials Register with the EudraCT number 2009-016923-77 (https://www.clinicaltrialsregister.eu/ctr-search/search?query=SURVET). Independent contract research organizations monitored the study and collected and maintained the data. The Department of Pharmaceutical Sciences of the University of Milan (Milan, Italy) analyzed the data. Each study center initiated the trial only after the local Ethics Committee or Institutional Review Board had approved the protocol. The study was performed in accordance with the protocol, with the Declaration of Helsinki, with Good Clinical Practice, and with local regulations.

The Steering Committee had final responsibility for verification and analyses of the data, wrote the manuscript, and vouches for the accuracy and completeness of the reported data. All authors contributed to the interpretation of the results, approved the final version of the manuscript, and made the decision to submit the manuscript for publication. The study was supported by Alfa Wassermann SpA (Via Ragazzi del 99, 5-Bologna, Italy), which supplied its commercially available capsules of sulodexide and manufactured the matching placebo. A separate, independent contract organization prepared the randomization list and the treatment units. Alfa Wassermann funded the study but played no role in the design of the study, in data collection or analysis, or in manuscript preparation.

### Statistical Analysis

Assuming an incidence of recurrent VTE with standard care of ≈17.5% in 2 years^3–7^ and hypothesizing a 50% relative reduction by adding sulodexide,^18^ we determined that a total of 620 patients (≈310 per group) had 90% power to show superiority of sulodexide over placebo at a 2-sided level of α=0.05.

The primary efficacy analysis, which considered all outcome events occurring from randomization to the end of treatment, was performed according to the intention-to-treat (ITT) principle and included all patients who had been randomized (except 2 blinded administrative exclusions). Hazard ratios, 95% confidence intervals (CIs), and *P* values were calculated with the Cox proportional hazards models and SPSS statistical software, version 17.0, with treatment as the only covariate. A Cox proportional hazards model analysis was also performed with adjustment for age (in decades), sex, type of index event (pulmonary embolism or deep vein thrombosis), country, dichotomized (<6/≥6 months) exposure to VKA, and dichotomized (<1/≥1 month) delay between the end of VKA treatment and randomization. An “all failures” efficacy analysis was performed in which all patients for whom no information on health status at 24 months was available were considered as having had an event (failure), the proportions of failures were compared by the Fisher exact probability test, and the incidence risk ratio and 95% CI were estimated with “epiR”^28^ in R.^29^ The outcome for patients lost to follow-up was also estimated by assigning the outcome of the nearest neighbor estimated by propensity score, computed from the same predictors as for the Cox regression except treatment. An additional sensitivity analysis was performed on the per-protocol population that included all patients of the ITT population who had the 24-month evaluation, had taken at least 75% of the planned study medication, and were exempt of major protocol violations as indicated by the study Steering Committee in a blind review. The safety analysis included all randomized patients.

## Results

### Patients and Study Treatment

**Figure 1.**
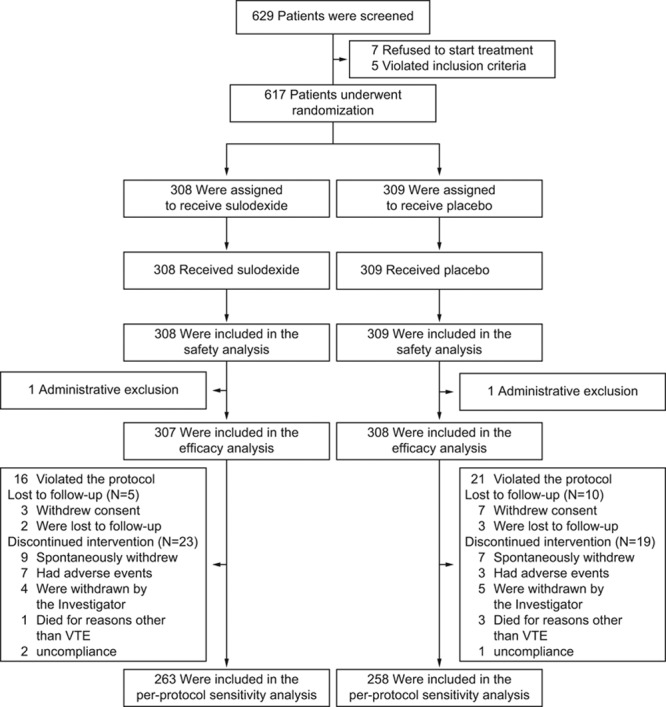
Enrollment and randomization. VTE indicates venous thromboembolism.

Between September 2010 and May 2012, 629 patients were screened in 43 centers in 7 European countries. The follow-up was closed on May 2014. Twelve patients were screening failures; 617 were included in the safety population. Two patients were excluded from efficacy analysis because of administrative reasons: 1 was the sole individual recruited in 1 of the planned countries, and 1 entered twice in the trial at 2 different sites, and the first entry was excluded from efficacy analysis. A total of 308 patients received placebo and 307 received sulodexide for a median duration of 23.9 months. The blinded review by the study Steering Committee included 521 patients in the per-protocol analysis (Figure 1). The study drug was discontinued prematurely in 28 patients given sulodexide (9.1%) and in 29 patients given placebo (9.4%; Figure 1). There were no significant differences between groups in baseline characteristics of the patients (Table 1), except for exposure to VKA (slightly more sulodexide patients in the <6-month category; *P*=0.044).

**Table 1.**
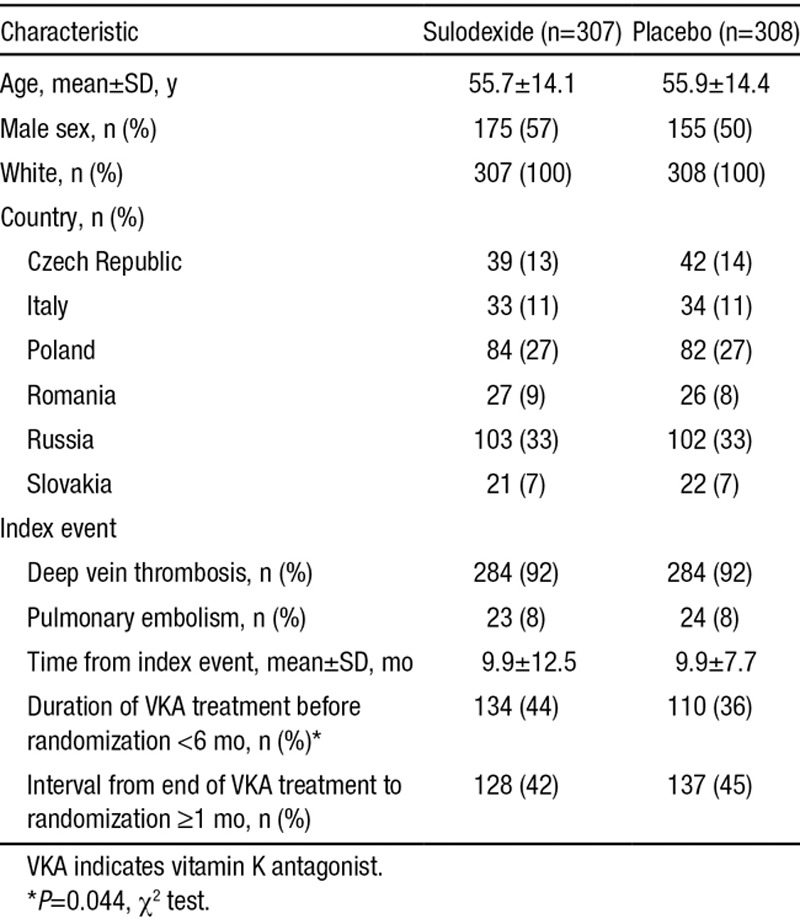
Demographic and Clinical Characteristics of the Patients According to Study Group

### Recurrent VTE

Recurrence of VTE occurred in 45 patients as a result of proximal deep vein thrombosis in 36 patients and pulmonary embolism in 9 patients (fatal in 1 patient).

**Figure 2.**
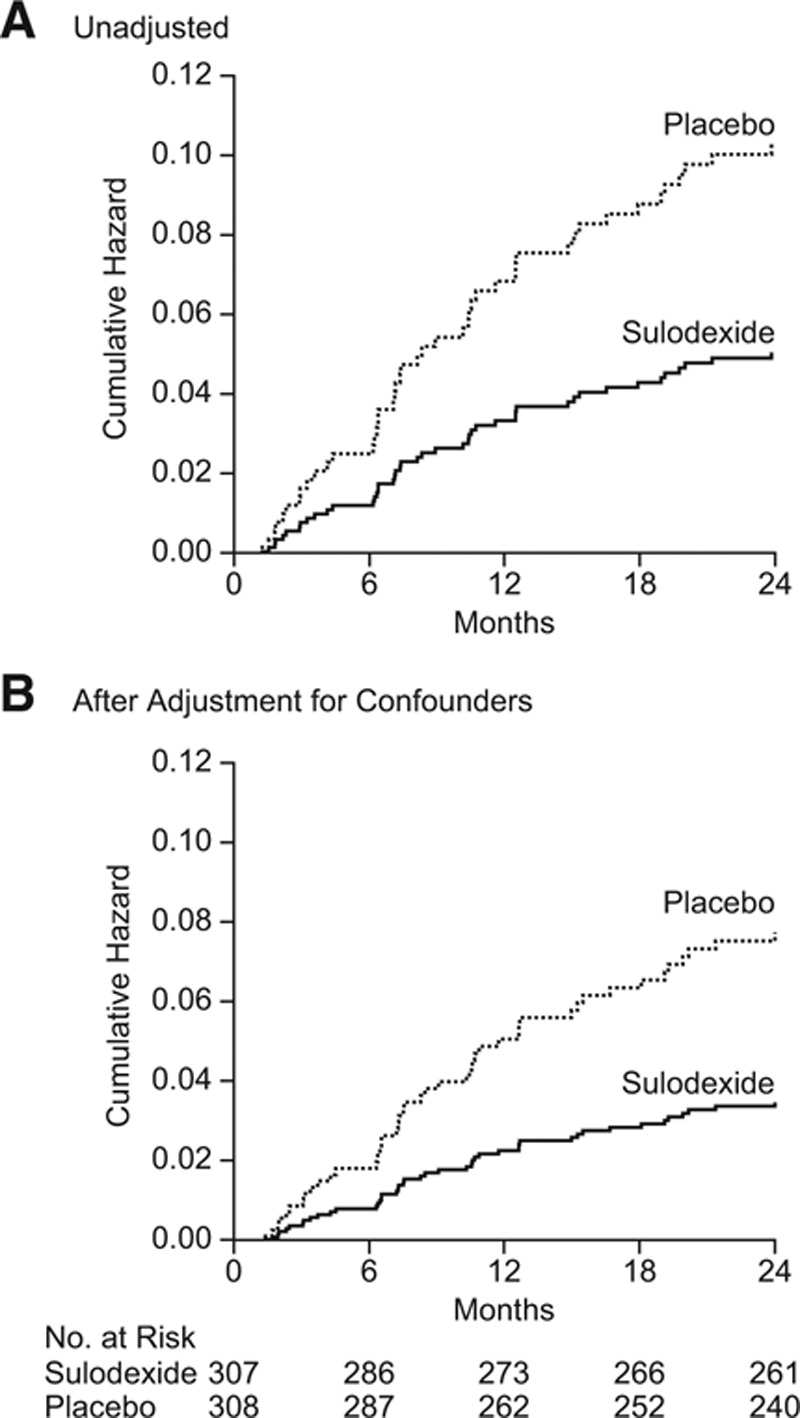
Risk of recurrence of venous thromboembolism in patients randomly assigned to sulodexide or placebo. **A**, Cumulative risk of recurrent venous thromboembolism. **B**, Results of an analysis of risk after adjustment for age, sex, index event (pulmonary embolism, or deep vein thrombosis), duration of anticoagulant therapy, and time from completion of anticoagulation therapy to randomization.

The primary outcome, recurrence of VTE, occurred in 15 of the 307 patients who received sulodexide (4.9%; 95% CI, 2.9–8.1) compared with 30 of the 308 patients who received placebo (9.7%; 95% CI:, 6.8–13.7; hazard ratio, 0.49; 95% CI, 0.27–0.92; *P*=0.02; Figure 2A).

The analysis adjusted for age, sex, index event (pulmonary embolism or deep vein thrombosis), country, duration of exposure to VKA, and delay between the end of VKA treatment and randomization confirmed that sulodexide treatment reduced the risk of recurrence (adjusted hazard ratio, 0.45; 95% CI, 0.24–0.84; *P*=0.01; Figure 2B). Independent risk factors for recurrent VTE included age (hazard ratio, 1.33 per decade; 95% CI, 1.06–1.65; *P*=0.01) and male sex (hazard ratio, 2.45; 95% CI, 1.25–4.78; *P*=0.01). No association was found between recurrent VTE and length of exposure to VKA (hazard ratio, 0.79; 95% CI, 0.41–1.53; *P*=0.48), delay between the end of VKA treatment and randomization (hazard ratio, 0.71; 95% CI, 0.37–1.36; *P*=0.71), country (*P*=0.09), or index event (hazard ratio, 1.67; 95% CI, 0.63–4.44; *P*=0.30).

Under the “all failures” assumption, the proportion of failures among control subjects was 48 of 308 or 15.6% (95% CI, 11.7–20.1) and that among treated patients was 26 of 307 or 8.5% (95% CI, 5.6–12.2; *P*=0.009, Fisher test). The incidence risk ratio of failure among treated patients was 0.54 (95% CI, 0.35–0.85) versus control subjects. The results of the logistic analysis adjusted for the same confounders indicated for the Cox analysis are reported in the text and in Table I in the online-only Data Supplement.

Applying the nearest-neighbor outcome to the 29 patients lost to follow-up using the propensity score yielded a proportion of events of 30 of 308 (9.7%) among control subjects and 16 of 307 (5.2%) among treated subjects (*P*=0.045, Fisher test; incidence risk ratio, 0.54; 95% CI, 0.30–0.96).

In the per-protocol population, VTE recurred in 14 of the 263 patients who received sulodexide compared with 30 of the 258 patients who received placebo (hazard ratio, 0.45; 95% CI, 0.24–0.85; *P*=0.014). In addition, the results of the adjusted Cox analysis in the per-protocol population did not differ appreciably from those in the ITT population (data reported in the online-only Data Supplement). The different procedures used to estimate the outcome in the ITT population resulted in a number needed to treat ranging 15 to 24, with variable width of the CI. The number needed to treat estimated from the adjusted Cox regression was 24 (95% CI, 16–98; details given in the online-only Data Supplement).

We also performed an unplanned subgroup analysis of recurrence rates by major potentially prognostic subgroups that failed to indicate subgroups more or less likely to respond to treatment (details in the text and Figure I in the online-only Data Supplement).

### Hemorrhagic Complications

There were no episodes of major bleeding. Clinically relevant, nonmajor bleeding occurred in 2 patients who received sulodexide (occasional nose bleeding in 1 patient, and 2 episodes of bleeding after evacuation in the other) and in 2 patients who received placebo (occasional events of rectal bleeding in 1 patient, and a dysfunctional uterine bleeding in the other). The hazard ratio for clinically relevant bleeding was 0.97 (95% CI, 0.14–6.88; *P*=0.98).

### Secondary End Points

Individually, none of the protocol-defined secondary end points was frequent enough to warrant a separate analysis (details in the online-only Data Supplement). The total incidence of primary plus secondary vascular events was 43 of 308 (14.0%; 95% CI, 10.3–18.3) among control subjects and 22 of 307 (7.2%; 95% CI, 4.5–10.6) among treated subjects (P=0.008, Fisher test; Table 2). Death occurred in 1 patient in the sulodexide group (as a result of stroke) and 3 patients in the placebo group (1 as a result of lower-limb ischemia, and 2 resulting from acute coronary syndrome).

**Table 2.**
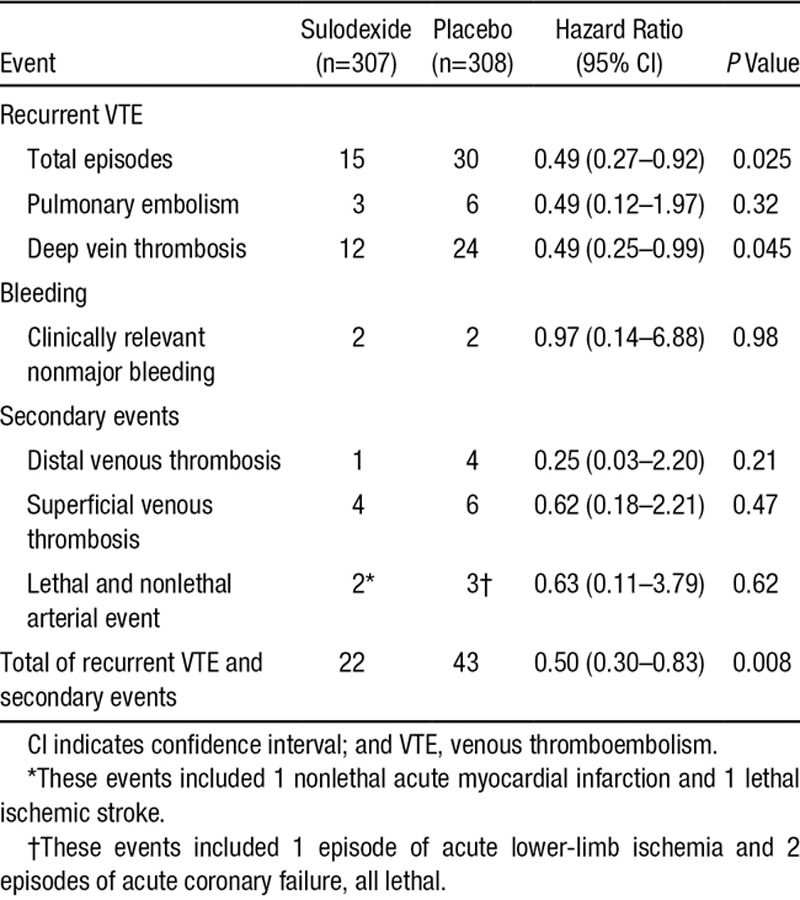
Number of Outcome Events According to Study Group

### Safety End Points

We analyzed the AEs in the safety data set. The 309 control and 308 treated patients reported 397 and 368 treatment-emergent AEs, respectively. There was no significant difference in the number of patients with at least 1 AE (52.4% of control versus 48.7% of treated subjects), at least 1 serious AE (11.0% versus 8.1%), at least 1 AE causing discontinuation (13.6% versus 9.1%), at least 1 AE resulting in death (1.3% versus 0.3%), and at least 1 not definitely unrelated AE (12.9% versus 16.6%). The most frequent (>1% of patients) AEs, regardless of the potential correlation with treatment, are reported in Table II in the online-only Data Supplement.

## Discussion

This study aimed at assessing whether a standard oral treatment with sulodexide after an anticoagulant regimen could, in addition to compression therapy, decrease the risk of recurrent deep vein thrombosis or pulmonary embolism over a period of 2 years.

The hazard ratio of qualifying events with sulodexide was 0.45 (95% CI, 0.24–0.84; *P*=0.01) after adjustment for age, sex, type of index event, country, exposure to VKA, and delay between the end of VKA treatment and randomization. Similar results were seen in the per-protocol population, in the “all failures” approach to the ITT population, and in the sensitivity analysis by propensity score in the ITT population.

The generalizability of these results appears sufficiently supported. The study included patients from different European countries with different healthcare systems without showing statistically significant heterogeneity.

The results of the SURVET study were similar to those of the trials performed with aspirin, the Warfarin and Aspirin (WARFASA) trial^30^ and the Aspirin to Prevent Recurrent Venous Thromboembolism (ASPIRE) trial,^31^ which were published while the SURVET study was underway. The pooled ASPIRE-WARFASA hazard ratio for VTE was 0.68 (95% CI, 0.51–0.90)^31^; the unadjusted hazard ratio in SURVET was 0.49 (95% CI, 0.27–0.92). The pooled ASPIRE-WARFASA hazard ratio for major vascular events was 0.66 (95% CI, 0.51–0.86) and that in SURVET was 0.50 (95% CI, 0.30–0.83). Finally, the ASPIRE-WARFASA pooled hazard ratio for clinically relevant bleeding was 1.47 (95% CI, 0.70–3.08) and that in SURVET was 0.97 (95% CI: 0.14–6.88). The studies performed with the newer direct anticoagulants, similarly published while the SURVET study was in progress, reported high efficacy compared with placebo for preventing recurrence (1.7% versus 8.8% with apixaban, 0.4% versus 5.6% with dabigatran, and 1.3% versus 7.1% with rivaroxaban) at the expense of increased major or clinically relevant nonmajor bleeding (3.2% versus 2.3%, 5.3% versus 1.8%, and 6.0% versus 1.2%, respectively).^10,13,14^

Our study, however, has some limitations. The total incidence of qualifying events was less than expected but similar to that of other trials.^32,33^ A better preventive approach during the period immediately after the index events and perhaps more frequent application of compressive therapy in the studied population could have contributed to decrease this incidence that, however, under the “all failures” assumption was close to the one anticipated in the sample size calculation. The smaller incidence of primary end point therefore appears unlikely to have biased the estimate of the effect size.

The proportion of patients entered in the study with major protocol violations was larger than expected. These violations included cases at lesser (longer anticoagulant treatment or short interval from anticoagulant withdrawal to randomization) and at higher (shorter or no anticoagulant treatment or long untreated interval before randomization) risk. None of these factors significantly affected the risk of recurrence in the multivariable analysis. Furthermore, the results in the per-protocol population were similar to those in the ITT population. There is therefore no evidence that the potential bias associated with protocol violations may have affected the estimate of the effect to an appreciable extent.

The proportion of patients prematurely interrupting the study without having reached the end point was also higher than expected yet limited for a 2-year study (5% total; 18 of 308 among control subjects and 11 of 307 among treated subjects). We performed a number of sensitivity analyses to monitor whether, and in which direction, this could have affected the assessment of the effect size. Applying constant risks ranging from 0 (“all successes” case) to 1 (“all failures” case) to the patients lost to follow-up yielded risk ratios from 0.50 (95% CI, 0.28–0.91; *P*=0.029) to 0.54 (95% CI, 0.35–0.85; *P*=0.009). Assigning instead the outcomes at random resulted in 228 possible combinations, with a median value of *P*=0.016. Not statistically significant results could occur only if the risk ratio of having the event among those randomized to treatment and lost to follow-up versus those randomized to control and lost to follow-up was ≥1.5. It was considered clinically improbable that patients extracted from a group who, when monitored, had a risk ratio of 0.49 (15 of 296 versus 30 of 290) could exhibit a risk ratio of ≥1.5 when not monitored. Finally, we performed a number of sensitivity analyses applying the nearest-neighbor outcome to the patients lost to follow-up using the propensity score, which was considered essentially independent from any assumption and more clinically reliable (more details are given in the online-only Data Supplement). These analyses yielded risk ratios between 0.44 (95% CI, 0.22–0.86; *P*=0.014) and 0.54 (95% CI, 0.30–0.96; *P*=0.045). The combination of the results of the survival analysis, those under the “all failures” assumption, those estimated by sensitivity analyses (in particular by propensity score), and those estimated per protocol, all comparable to each other, suggests that the subjects who left the study prematurely were a random subset of the total population and that the estimates of the effect size were sufficiently accurate for all practical purposes.

The proportion of patients with pulmonary embolism as the index event was low (7.6%). The results of this study should therefore be considered poorly applicable to this specific subpopulation.

Safety was favorable without unexpected AEs, likely in correlation with the treatment and clinically irrelevant risks of bleeding despite the 2-year continued treatment. It should be noted, however, that the absence of serious bleeding could be a chance finding because this study was underpowered to detect events occurring with very small frequency.

### Conclusions

Treatment with oral sulodexide at 500 lipasemic units twice daily for 2 years along with compression therapy decreased the incidence of recurrences of thromboembolic events without detectable risks for the patient safety. Future investigations should examine whether a similar effect can be obtained after treatment of the index event with non-VKA oral anticoagulants; whether there is a summation of effects with aspirin; whether prevention of recurrence could equally be performed with sulodexide, antiplatelets, or extended anticoagulation; and whether specific subgroups are more or less likely to benefit from sulodexide or other treatments.

## Acknowledgments

We are indebted to all the patients who agreed to participate in this study and to all clinical centers (listed in the online-only Data Supplement) that contributed to recruiting the patients. Study Committee: The SURVET study was monitored by a Steering Committee of 3 experts: Drs Andreozzi, Davì, and Palareti. The same committee, having full blinded access to all the data, acted also as Adjudication Committee to define the occurrence of events and the attribution of individual subjects to the analysis populations, after which the database was frozen. The same committee monitored the statistical analysis, which was performed blinded by the study statistician (Dr Bignamini) on the frozen database.

## Sources of Funding

The study was financed by Alfa Wassermann, manufacturer of sulodexide.

## Disclosures

Dr Andreozzi received consultancy fees or lecture grants from Mediolanum Farmaceutici, Alfa Wassermann, and Laboratorios Elmor. Drs Bignamini and Davì received consultancy fees from Bayer Healthcare and Alfa Wassermann. Dr Palareti received consultancy fees from Alfa Wassermann and Daiichi-Sankyo, as well as lecture fees from Werfen Group and Stago. Dr Sokurenko received lecture grants from Alfa Wassermann and Sanofi. The other authors report no conflicts.

## Supplementary Material

## CLINICAL PERSPECTIVE

Patients with unprovoked venous thromboembolism are at high risk for recurrence after discontinuation of treatment with vitamin K antagonists (VKAs). Extending treatment with VKAs reduces the recurrence risk but increases the bleeding risk. In clinical practice, VKAs are generally discontinued when the perceived risk of bleeding outweighs the risk of recurrence. Drugs with low or no bleeding risk and less aggressive antithrombotic activity may represent adequate alternatives to continue anticoagulation with VKAs, or patients should be left to only physical management (elastic stockings) in cases of doubt. Rates of bleeding in general inferior to VKAs and efficacy not inferior to VKAs have been shown by the newer non-VKAs. However, compared with placebo, the extended anticoagulation with dabigatran, rivaroxaban, or apixaban, although reducing the risk of venous thromboembolism recurrence, carried a higher risk of major or clinically relevant nonmajor bleeding. The pooled data of the Warfarin and Aspirin (WARFASA) and Aspirin to Prevent Recurrent Venous Thromboembolism (ASPIRE) trials showed a significant risk reduction of venous thromboembolism recurrence, although at a lower extent than with the new non-VKAs, but still a worse result than placebo in terms of the occurrence of clinically relevant bleeding. In the 2 years of treatment in the Sulodexide in Secondary Prevention of Recurrent Deep Vein Thrombosis (SURVET) study, venous thromboembolism recurred in 15 of 307 patients on sulodexide and 30 of 308 on placebo (hazard ratio, 0.49; 95% confidence interval, 0.27–0.92; *P*=0.02). There were no differences in major or clinically relevant nonmajor bleeding between the sulodexide and placebo groups. Sulodexide appears to be an important treatment option when extended anticoagulation is potentially useful but associated with unwanted bleeding risk.
