# Supplementary material for: Sulodexide for the Prevention of Recurrent Venous Thromboembolism: The Sulodexide in Secondary Prevention of Recurrent Deep Vein Thrombosis (SURVET) Study: A Multicenter, Randomized, Double-Blind, Placebo-Controlled Trial
Source: Circulation. 2015 Nov 16;132(20):1891–7. doi: 10.1161/CIRCULATIONAHA.115.016930 (PMC4643750; doi:10.1161/CIRCULATIONAHA.115.016930)
Supplement: Supplementary file 1 [file cir-132-1891-s001.docx]

Patients with unprovoked venous thromboembolism are at high risk for recurrence after discontinuation of treatment with vitamin-K antagonists (VKAs). Extending treatment with VKAs reduces the recurrence risk but increases the bleeding risk. In clinical practice, VKAs are generally discontinued when the perceived risk of bleeding outweighs the risk of recurrence. Drugs with low or no bleeding risk and less aggressive antithrombotic activity may represent adequate alternative to continue anticoagulation with VKAs or to leave patients to only physical management (elastic stockings) in cases of doubt. Rates of bleeding in general inferior to VKAs and efficacy not inferior to VKAs have been shown by the newer non-VKAs. However compared to placebo, the extended anticoagulation with dabigatran, rivaroxaban or apixaban while reducing the risk of VTE recurrence, carried out a higher risk of major or clinically relevant nonmajor bleeding. The pooled data of the aspirin’ WARFASA and ASPIRE trials, showed a significant risk reduction of VTE recurrence, although at a lower extent than the new non-VKAs, but still a worse result than placebo as regards the occurrence of clinically relevant bleeding. In the 2 years’ treatment of the SURVET study, VTE recurred in 15 of 307 patients on sulodexide and 30 of 308 on placebo (hazard ratio, 0.49; 95% confidence interval 0.27 to 0.92; P=0.025). There were no differences in major or clinically relevant nonmajor bleeding between sulodexide and placebo groups. Sulodexide appears to be an important treatment option when extended anticoagulation is potentially useful but associated with unwanted bleeding risk.

(249 words)
