# Supplementary material for: Sulodexide for the Prevention of Recurrent Venous Thromboembolism: The Sulodexide in Secondary Prevention of Recurrent Deep Vein Thrombosis (SURVET) Study: A Multicenter, Randomized, Double-Blind, Placebo-Controlled Trial
Source: Circulation. 2015 Nov 16;132(20):1891–7. doi: 10.1161/CIRCULATIONAHA.115.016930 (PMC4643750; doi:10.1161/CIRCULATIONAHA.115.016930)
Supplement: Supplementary file 2 [file cir-132-1891-s002.pdf]

SUPPLEMENTAL MATERIAL

Sulodexide for the Prevention of Recurrent Venous Thromboembolism: The SURVET Study: A Multicenter, Randomized, Double-Blind, Placebo Controlled Trial

SUPPLEMENTAL RESULTS

Results of the logistic analysis assigning all lost to follow-up to failure

All patients without confirmed information as to the health status at 24 months after randomization were classified as failures, as if they had had reached the endpoint (recurrence of thromboembolism). All patients with confirmed recurrent thromboembolism were also classified as failure. Only patients with definite information that in the 24-month period after randomization had not had the event were classified as success.

The logistic regression analysis adjusted the results observed by treatment, for sex, age, length of exposure to VKA (<6 months/≥6months), delay from the end of VKA treatment and randomization (<1 month/≥1 month), country, and type of index event (deep vein thrombosis/pulmonary embolism).

The model resulted to fit well (Hosmer and Lemeshow test: P=0.620) and to improve by almost 10% the accuracy of prediction over the null model (Nagelkerke R square=0.098).

The results confirmed that also in terms of failure under the worst-case assumption, the significant predictors were the same as those indicated by the Cox analysis (Table S-1).

Proportion of events assigning the outcome to patients lost to follow-up by propensity score

A sensitivity analysis of the outcome was also performed by assigning the outcome to the patients lost to follow-up by propensity score.

If we assume that the risk of recurrence among those who abandoned the study is determined by the factors considered putative predictors of the event - with the exclusion of treatment – we can estimate the propensity score for recurrence from the monitored patients. From the relevant equation, we can estimate the score for those lost to follow-up; subsequently the patients lost to follow-up are assigned the status (event/no-event) of the nearest neighbor.

We estimated the propensity score for having the primary event using the data from the 586 patients who either had the event or reached the 24 months without event. As predictors, the same used for the Cox survival analysis were employed, once considering treatment and once not considering treatment.

The equations estimating the propensity score were then applied to the 29 patients lost to follow-up.

CASE: CONSIDERING TREATMENT IN THE EQUATION

The 29 patients lost to follow-up were assigned the outcome exhibited by the subject of the same treatment group, having the nearest propensity score. This assigned 1 case among placebo and none among treated to the category FAILURE. The resulting estimate of the proportion of events was 31/308 (10.1%) among controls, and 15/307 (4.9%) among treated (P=0.021, Fisher's test; incidence risk ratio: 0.49 [0.27-0.88]).

We repeated the same procedure, assigning to the 29 cases the outcome exhibited by the subject with the nearest propensity score, regardless of the treatment group. This assigned 2 cases among placebo and none among treated to the category FAILURE. The resulting

estimate of the proportion of events was 32/308 (10.4%) among controls, and 15/307 (4.9%) among treated (P=0.014, Fisher's test; incidence risk ratio: 0.44 [0.22-086]).

CASE: NOT CONSIDERING TREATMENT IN THE EQUATION

The 29 cases were assigned the outcome exhibited by the subject with the nearest propensity score, regardless of the treatment group. This assigned 0 cases among placebo and 1 among treated to the category FAILURE.

The resulting estimate of the proportion of events was 30/308 (9.7%) among controls, and 16/307 (5.2%) among treated (P=0.045, Fisher's test; incidence risk ratio: 0.54 [0.30-0.96]).

Regardless of the approach taken, the results consistently confirmed that the probability of having a recurrence of DVT/PE was significantly greater among controls than among treated patients.

The variations that could be seen with the different procedures to assign outcomes to the patients lost to follow-up affected the size, but not the direction, of the effect.

NNT estimates for the primary clinical endpoint (recurrence of DVT)

We estimated the NNT to avoid one event more of recurrent DVT/PE in two years with the indicated dosage scheme of sulodexide added to the standard of care, vs. the standard of care alone. Since the probability of recurrence was estimated under different assumptions and with different techniques, several different estimates of NNT were computed.

ESTIMATES FROM THE ABSOLUTE RISK REDUCTION

The most common estimate of NNT is from the absolute risk reduction that, however, in this study should be estimated under the different assumptions made about the cases lost to follow-up.

1. The estimate from the absolute risk reduction (considering all lost to follow-up as non-events) yielded NNT=21 [95% CI: 10-232].
2. The estimate from the absolute risk reduction (considering all lost to follow-up as events) yielded NNT=15 [95% CI: 7-60].
3. The estimate from the absolute risk reduction (excluding all lost to follow-up) yielded NNT=19 [95% CI: 10-159].

However, these estimates do not take into account neither the actual exposure to treatment, nor the effect of potential confounders that, even in a randomised study, is definitely evident (as shown by the significant effects of predictors at the Cox analysis). We therefore estimated the NNT from the Kaplan-Meyer procedure, the unadjusted NNT from the Cox regression analysis and the NNT from the adjusted Cox regression analysis (using the covariates indicated in the text). (Altman DG, Andersen PK. Calculating the number needed to treat for trials where the outcome is time to an event. BMJ. 1999;319:1492-1495)

ESTIMATES FROM SURVIVAL ANALYSES

4. The estimate from the Kaplan-Meyer was NNT = 19 [95% CI: 10-102].

5. The estimate from the unadjusted Cox regression was NNT = 20 [95% CI: 13 - 121].

6. The estimate from the adjusted Cox regression was NNT = 24 [95% CI: 16-98].

Overall, while the NNT is approximately 20, the width of the confidence interval is largely determined by the application of adjustments for exposure to treatment (that, being longer for the treated group, reduces the point estimate of the NNT) and for the potential confounders (which results in substantially smaller width of the confidence interval). Under actual clinical conditions, the NNT estimated from the adjusted Cox regression of 24 [16-98] can be considered to reflect the true treatment effect.

Further studies, which will allow estimating the NNT from the summary measure of effect, would allow to better estimate the point NNT and to reduce the width of the confidence interval.

Results in the per-protocol population

The per-protocol population was composed of 521 patients, of whom 263 received sulodexide and 258 received placebo (Figure 1). Venous thromboembolism recurred in 44 patients (one patient with a primary event was excluded from this population because of a major protocol violation) and was due to deep-vein thrombosis in 36 patients and to pulmonary embolism in 8 patients (fatal in 1 patient).

The primary outcome, recurrence of venous thromboembolism, occurred in 14 of the 263 patients who received sulodexide, as compared with 30 of the 258 patients who received placebo (hazard ratio, 0.45; 95% CI, 0.24 to 0.85; P = 0.014).

The analysis adjusted for age, sex, index event (pulmonary embolism or deep-vein thrombosis), country, duration of exposure to VKA, and delay from end of VKA treatment and randomization, confirmed that sulodexide treatment reduced the risk of recurrence (adjusted hazard ratio, 0.43; 95% CI, 0.23 to 0.81; P = 0.01). Independent risk factors for recurrent venous thromboembolism included age (hazard ratio, 1.03; 95% CI, 1.01 to 1.05; P = 0.02), male sex (hazard ratio, 2.40; 95% CI, 1.23 to 4.70; P = 0.01), and marginally the country (P=0.042 without any country differing significantly from the overall trend). No association was found between recurrent venous thromboembolism and length of exposure to VKA (hazard ratio, 0.84; 95% CI, 0.43 to 1.68; P = 0.63), delay from end of VKA treatment and randomization (hazard ratio, 0.71; 95% CI, 0.37 to 1.38; P = 0.31), or index event (hazard ratio, 1.74; 95% CI, 0.65 to 4.64; P = 0.27).

Unplanned subgroup analysis of the incidence of primary events

We estimated the risk ratio of recurrence in different subgroups of potential prognostic relevance, after exclusion of the cases lost to follow-up. The analysis was performed with epiR in R. No formal comparison was performed across subgroups, since the 95% confidence intervals are already sufficient to estimate the extent of superposition across levels of subgroups, and the displacement of the individual estimate from the overall estimate of the effect.

This unplanned subgroup analysis was performed with the exclusive aim of detecting whether there was any major discrepancy across potentially important subgroups, that could suggest major modifications to protocol in future randomized controlled trials. Indeed, being the analysis unplanned, any possible difference seen by subgroups levels, could only be considered a hypothesis-generating finding.

The results are summarized in Figure S-1.

Secondary vascular events

Five patients had distal leg DVT (4 randomized to placebo *vs.* 1 randomized to sulodexide), 10 had superficial vein thrombosis (6 *vs.* 4), and 5 had arterial events considered secondary endpoints (3 *vs.* 2). The incidence of these events did not differ between groups, although each of these events occurred more frequently among controls. The number of patients who had any one of these secondary events was 13/308 among the patients randomized to placebo, and 7/307 among those randomized to sulodexide (4.2% vs. 2.3%), without evidence of a significant difference (P=0.26).

Some arterial events were considered secondary study endpoint (AMI, stroke, peripheral ischemia); others were not (identification of carotid stenosis or peripheral artery thrombosis). Overall, 9/308 patients among controls exhibited arterial events (2.9%; 95% CI: 1.3-5.5%) *vs.* 4/307 among treated patients (1.3%; 95% CI: 0.4-3.3%; P=0.262, Fisher test). The IRR with sulodexide was comparable with that observed for the occurrence of venous events: 0.45 [0.14-1.43].

SUPPLEMENTAL TABLES

Table S-1. Odds ratio (OR) for the putative predictors in the multivariable logistic analysis of failures under the worst-case scenario.

Predictor	OR [95% confidence interval]	P
Treatment: sulodexide	0.467 [0.277-0.787]	0.004
Male sex	1.837 [1.083-3.116]	0.024
Age	0.979 [0.961-0.997]	0.024
Exposure to VKA ≥6months	0.855 [0.495-1.478]	0.574
Randomization ≥1 month after the end of VKA treatment	0.830 [0.479-1.439]	0.507
Country*		0.127
Index_event: pulmonary embolism	1.251 [0.512-3.057]	0.624

* none of the countries deviated significantly from the overall trend

Table S-2. Number of Patients with Adverse Events and Number of Adverse Events by Study Groups.*

	Sulodexide (N=308)	Placebo (N=309)
regardless of correlation		
any	150 (48.7) [368]	162 (52.4) [397]
severe	22 (7.1) [35]	25 (8.1) [36]
causing treatment interruption	28 (9.1) [31]	42 (13.6) [48]
serious	25 (8.1) [30]	34 (11.0) [45]
causing death	1 (0.3) [1]	4 (1.3) [5]
potentially correlated		
any	51 (16.6) [94]	40 (12.9) [77]
severe	7 (2.3) [10]	6 (1.9) [9]
causing treatment interruption	13 (4.2) [14]	12 (3.9) [13]
serious	9 (2.9) [11]	5 (1.6) [7]
causing death	0 (0.0)	0 (0.0)
most frequent (>1%), regardless of correlation		
Pain in extremity	15 (4.9) [23]	16 (5.2) [19]

Deep vein thrombosis	12 (3.9) [13]	24 (7.8) [24]
Arthralgia	13 (4.2) [20]	8 (2.6) [10]
Hypertension	10 (3.2) [11]	13 (4.2) [13]
Oedema peripheral	13 (4.2) [13]	6 (1.9) [8]
Nasopharyngitis	11 (3.6) [11]	8 (2.6) [10]
Respiratory tract infection viral	9 (2.9) [9]	7 (2.3) [8]
Headache	2 (0.6) [2]	11 (3.6) [15]
Pulmonary embolism	5 (1.6) [5]	8 (2.6) [8]
Vertigo	5 (1.6) [6]	5 (1.6) [7]
Diarrhoea	5 (1.6) [5]	7 (2.3) [7]
Abdominal pain upper	6 (1.9) [6]	5 (1.6) [5]
Urinary tract infection	1 (0.3) [1]	9 (2.9) [9]
Hypercholesterolaemia	6 (1.9) [8]	2 (0.6) [2]
Blood pressure increased	1 (0.3) [2]	7 (2.3) [7]
Back pain	2 (0.6) [2]	5 (1.6) [7]
Blood glucose increased	5 (1.6) [5]	3 (1.0) [3]
Gout	4 (1.3) [5]	3 (1.0) [3]
Pain	4 (1.3) [4]	2 (0.6) [3]
Dyspnoea	4 (1.3) [4]	2 (0.6) [3]

Upper respiratory tract infection	3 (1.0) [3]	4 (1.3) [4]
Bronchitis	3 (1.0) [3]	4 (1.3) [4]
Sciatica	2 (0.6) [2]	4 (1.3) [4]
Pruritus	4 (1.3) [5]	1 (0.3) [1]
Nausea	0 (0.0) [0]	4 (1.3) [6]
Carotid arteriosclerosis	1 (0.3) [2]	4 (1.3) [4]
Vomiting	0 (0.0) [0]	5 (1.6) [6]
Condition aggravated	5 (1.6) [5]	0 (0.0) [0]

* number of patients with the events (%). Square brackets denote the number of nonconsecutive events.

SUPPLEMENTAL FIGURES

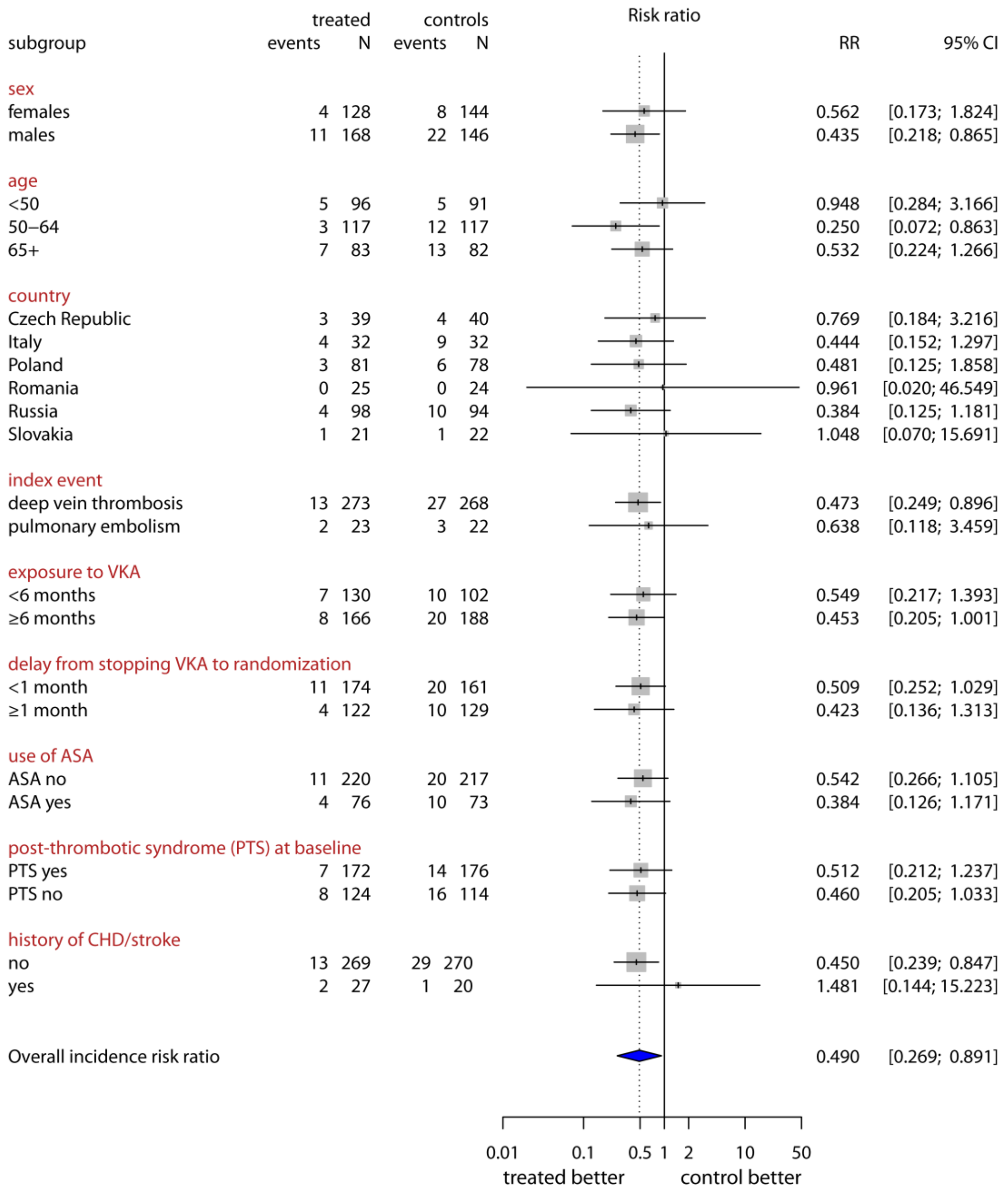

Supplemental Figure S-1. Unplanned analysis of the risk ratio for recurrent VTE (with 95% confidence interval) in the SURVET Study patients (after exclusion of the cases lost to follow-up), stratified by clinically relevant subgroups.

STUDY GROUP MEMBERS

Czech Republic: IKEM - Kardiologická klinika, Praha: *Karel Roztočil*; Fakultní nemocnice Plzeň - Interní oddělení, Plzeň – Bory: *Jana Hirmerova*; Angiologická ambulance - Horní Valy 13 - 69501 Hodonín: *Jiří Matuška*; Angiologická ambulance, Brno: *Václav Pecháček*; Nemocnice České Budějovice - Interní oddělení, České Budějovice: *Martin Holý*.

Poland: Gabinet Lekarski, Katowice: *Tomasz Urbanek*; Nzoz Perełka, Łódź: *Jacek Śmigielsk*i; Poradnia Chorób Naczyń Obwodowych MIKOMED, Łódź: *Jacek Mikosiński, Mirosla Wasiewicz, Tomasz Lesiak*; Specjalistyczny Gabinet Lekarski Chorób Tętnic i Żył, Lublin: *Piotr Niedziela*; Specjalistyczna Praktyka Lekarska, Giżycko: *Marek Sajkowski*; Life - Med. Lecznica, Grodzisk Mazowiecki: *Grzegorz Madycki*; ProfMedica, Poznań: *Katarzyna Pawlaczyk-Gabriel, Marcin Gabriel, Zbigniew Krasiński.*

Slovakia: ALIAN s.r.o., Bardejov: *Andrej Džupina*; Ústredná vojenská nemocnica - Interná klinika, Ružomberok: *Antonín Hruboň, Miroslav Urban*; VASA CARE, s.r.o. -Angiology Lab, Trnava: *Ivar Vacula*

Russian Federation: Moscow State Medical Institution «City Clinical Hospital № 1 n.a. Pirogov», Moscow: *Kirienko Alexander Ivanovich*; Clinical Hospital of Russian Academy of Sciences, St. Petersburg: *Balluzek Marina Feliksovna, Basos Sergey*; Municipal Medical Institution «City Emergency Care Hospital», Kursk: *Chernyatina Marina Aleksandrovna, Gladchenko Mikhail*; St. Petersburg State Medical Institution «City Multidisciplinary Hospital № 2», St. Petersburg: *Didenko Yury Pavlovich, Androsova Svetlana*; Municipal Medical Institution «City Hospital № 3», Chelyabinsk: *Fokin Alexey Anatolievich, Degtyarev Maxim*; Municipal Medical Institution «Clinical Hospital № 8, Yaroslavl», Yaroslavl: *Malygin Alexander Yurievich, Korzhova Valeria*; Municipal Medical Institution «Vsevolozhsk Central District Hospital», Vsevolozhsk: *Matevosyan Elena Nikolaevna, Vorontsova Tatiana*; St.

Petersburg State Medical Institution «City Hospital № 40 of Kurortny Administrative Region», St. Petersburg: Sarana Andrey Mikhailovich, Agafina Alina; St.Petersburg State Institution of Public Health «Consultive and Diagnostic Center # 85», St. Petersburg: S*okurenko German Yurievich, Popovich Vladimir*; Federal State Institution «National Medical Surgical Center n.a. Pirogov of Roszdrav», Moscow: *Stoyko Yuriy Mikhailovich, Linchak Ruslan*; Federal State Medical Institution «Clinical Hospital № 122 n.a. Sokolov of FMBA of Russia», St. Petersburg: *Svetlikov Alexei Vladimirovich, Gamzatov Temirlan*; State Medical Institution «Voronezh Regional Clinical Hospital № 1» Voronezh: *Esipenko Viktor Vasilievich*; State Medical Institution of Moscow «City Hospital № 13», Moscow: *Rodoman Grigory Vladimirovich, Zakhovskaya Elena*; Non-governmental Medical Institution «Road Clinical Hospital of public corporation «Russian Railways» St. Petersburg: *Sonkin Igor Nikolaevich, Melnik Valerii*.

Romania: CMI Dr Militaru, Craiova: *Constantin Militaru*; Quantum Medical Center, Bucharest: *Radu Dumitru Stoenescu*; C.M.Dr. Blaj Stefan, Bucharest: *Stefan Blaj*; Spitalul Clinic Judetean de Urgenta, Brasov: *Stefan Blaj*; Spitalul Clinic Judetean de Urgenta, Brasov: *Laurentia Doina Andrei*.

Portugal: Hospital Pulido Valente, Lisboa: *Albino Pereira*.

Italy: Dipartimento di Medicina e Scienze dell'Invecchiamento, Ce.S.I. Università "G.D'Annunzio" Chieti (Italy): *Giovanni Davì, Gianfranco Lessiani*; Angiology Care Unit, University Hospital, Padua: *Giuseppe Camporese, Romeo Martini, Chiara Tonello*; Presidio Ospedaliero di Castelfranco Veneto, Castelfranco Veneto: *Adriana Visonà, Laura Zotta*; U.O.S.D. di Angiologia - Medicina Interna - Ospedale SS. Giovanni e Paolo, Venezia: *Roberto Parisi*; Medicina Generale II - Azienda Ospedaliera di Vimercate, Vimercate: *Guido Giuseppe Arpaia, Gabriella Spezzigu*; Azienda Ospedaliera Complesso Osped. S. Giovanni

Addolorata, Roma: *Claudio Allegra*; U.O. Angiologia Azienda Ospedaliera Universitaria Policlinico P. Giaccone, Palermo: *Filippo Ferrara*, *Corrado Amato*; Azienda Ospedaliera Universitaria Ospedale Vittorio Emanuele e Ferrarotto, Catania: *Michelangelo Di Salvo*, *Giacomo Failla*.
